# Proguanil Suppresses Breast Tumor Growth In Vitro and In Vivo by Inducing Apoptosis via Mitochondrial Dysfunction

**DOI:** 10.3390/cancers16050872

**Published:** 2024-02-22

**Authors:** Nehal Gupta, Marina Curcic, Sanjay K. Srivastava

**Affiliations:** 1 Department of Biomedical Sciences, Texas Tech University Health Sciences Center, Amarillo, TX 79106, USA; nehal.gupta@ttuhsc.edu; 2 Department of Immunotherapeutics and Biotechnology, Jerry H. Hodge School of Pharmacy, Texas Tech University Health Sciences Center, 1718 Pine Street, Abilene, TX 79601, USA; mcurcic@ttuhsc.edu; 3 Center for Tumor Immunology and Targeted Cancer Therapy, Texas Tech University Health Sciences Center, 1718 Pine Street, Abilene, TX 79601, USA

**Keywords:** mitochondria, ROS, drug repurposing, oxidative phosphorylation, DNA damage, apoptosis, mitochondrial dysfunction, antimalarial drug, breast cancer

## Abstract

**Simple Summary:**

This study is focused on exploring the anticancer properties of proguanil, an old antimalarial drug. Proguanil demonstrated a significant ability to induce cell death through the increased production of reactive oxygen species (ROS) and subsequent disruption in mitochondrial function in breast cancer cells. In several breast cancer cell lines and mouse tumor models, proguanil showed promising results by inhibiting tumor growth and altering key proteins associated with cancer progression. These findings suggest that proguanil could be a promising candidate for further clinical investigation as a potential treatment option for breast cancer.

**Abstract:**

Breast cancer, ranking as the second leading cause of female cancer-related deaths in the U.S., demands the exploration of innovative treatments. Repurposing FDA-approved drugs emerges as an expedited and cost-effective strategy. Our study centered on proguanil, an antimalarial drug, reveals notable anti-proliferative effects on diverse breast cancer cell lines, including those derived from patients. Proguanil-induced apoptosis was associated with a substantial increase in reactive oxygen species (ROS) production, leading to reduced mitochondrial membrane potential, respiration, and ATP production. Proguanil treatment upregulated apoptotic markers (Bax, p-H2AX, cleaved-caspase 3, 9, cleaved PARP) and downregulated anti-apoptotic proteins (bcl-2, survivin) in breast cancer cell lines. In female Balb/c mice implanted with 4T1 breast tumors, daily oral administration of 20 mg/kg proguanil suppressed tumor enlargement by 55%. Western blot analyses of proguanil-treated tumors supported the in vitro findings, demonstrating increased levels of p-H2AX, Bax, c-PARP, and c-caspase3 as compared to controls. Our results collectively highlight proguanil’s anticancer efficacy in vitro and in vivo in breast cancer, prompting further consideration for clinical investigations.

## 1. Introduction

Breast cancer accounting for 30.8% of all cancers is prominent among women, ranking as the second leading cause of cancer-related mortalities in the U.S. [1]. It is a heterogeneous disease in which hormone disruption, alteration in oncogenic signaling pathways, protein expression deregulation, and genomic alterations take place [2]. Despite enhancements in the 5-year survival rate following diagnosis, the essential need to formulate innovative treatment approaches for breast cancer management is a priority. This will address improving treatment outcomes. Repurposing well-known and well-characterized non-cancer drugs for new uses in oncology is vital as it can save time and costs needed for drug development, and hence reach patients quicker [2].

Reactive oxygen species (ROS) are a group of reactive, unstable, and partially reduced oxygen derivatives, encompassing hydrogen peroxide (H_2_O_2_), superoxide anion (O^2−^), singlet oxygen (^1^O^2^), hydroxyl radical (**·**OH), and hypochlorous acid (HOCl) [3]. Cellular ROS can be induced endogenously, as in the process of oxidative phosphorylation, or may originate from contact with external sources, such as xenobiotics [4].

Oxidative stress, resulting from an imbalance due to an excess of ROS, is implicated in diverse disease conditions, including diabetes, atherosclerosis, cancer, neurodegeneration, and aging [5,6,7]. The relationship between ROS and cancer has been consistently debated. Some studies have indicated that ROS play a significant role in both the initiation and progression of cancer [8,9,10]. Conversely, many chemotherapeutic and radiotherapeutic agents induce the elimination of cancer cells by increasing ROS [11,12,13]. This implies that cancer cells can be killed by the same mechanism that promotes their survival; the answer lies in the level of ROS produced.

Proguanil, previously known as Paludrine, is an antimalarial agent that was developed in England during World War II. N1-p-chlorophenyl-N5-isopropylbiguanide was synthesized, and this compound demonstrated a high degree of activity against avian malaria [14]. Proguanil is used as a preventive and therapeutic agent for malaria both in adults and children. It is a prodrug, and its active metabolite is cycloguanil. Although both proguanil and cycloguanil inhibit malaria parasites, they have different targets [15]. Cycloguanil is an inhibitor of dihydrofolate reductase (DHFR), which is essential for DNA and amino acid synthesis for malaria parasites. Thus, inhibition of DHFR results in parasitic death [16]. Inhibition of DHFR by cycloguanil seems to be specific for the parasitic DHFR, as parasites transformed with human DHFR showed high resistance to this drug [17]. At the same time, the sensitivity of transformed and non-transformed parasites to proguanil was unchanged, indicating possible different targets of proguanil compared to its metabolite [18]. The literature suggests that proguanil has limited intrinsic activity and that its activity is mostly associated with mitochondrial function [18]; thus, its exact mechanism of action is not fully understood.

The combination of proguanil with atovaquone, sold under the brand name ‘Malarone’ [19], has been shown to be successful for chloroquine-resistant cases of malaria [20,21]. A study indicated that the addition of proguanil increases the ability of atovaquone to reduce membrane potential without having any effect on electron transport inhibition in parasites [22]. Proguanil by itself had minimal effects on the electron transport and membrane potential (ΔΨm) of the parasite [23]. On the other hand, as indicated in another study, proguanil’s synergy with atovaquone, the specific target of which is the bc1 complex, indicates the mitochondria as the location of proguanil’s activity [24].

Previously we have established the anticancer efficacy of atovaquone in primary, paclitaxel-resistant, and metastatic breast cancer [25]. Since the two compounds work in synergism, we further wanted to test the effects of proguanil in a breast cancer model.

Proguanil is unable to enter the mitochondrial matrix of mammalian cells, unlike its related biguanide phenformin [25]. However, it demonstrated a better anti-proliferative effect in cancer cells compared to other biguanides [26]. Several other studies confirmed the cytotoxic effects of proguanil in ovarian, bladder, and glioblastoma cancer cells, but its exact mechanism of action was not clear [23,27,28]. In the present study, we demonstrate that proguanil significantly inhibits the proliferation of human breast cancer cell lines (HCC1806, MCF-7, and MDA-MB-231) along with several patient-derived breast cancer cell lines. We show that the apoptosis-inducing effect of proguanil in these cells is related to the generation of ROS in the mitochondria, mitochondrial depolarization, and inhibition of mitochondrial respiration resulting in the activation of the caspase-3 apoptosis cascade. The in vitro findings were further corroborated by demonstrating 4T1 tumor size reduction by proguanil in an in vivo orthotopic breast tumor model.

## 2. Materials and Methods

### 2.1. Chemicals

The proguanil used in the study was 98.2% pure and obtained from Sigma Aldrich (Saint Louis, MO, USA). It was dissolved in DMSO where it was stable for an extended period of time. The bioavailability of proguanil when taken orally is typically 100% according to the literature [29]; however, some studies have reported lower values [30].

### 2.2. Cell Culture

Human breast carcinoma cell lines (MDA-MB-231, MCF-7, HCC1806) and a murine breast cancer cell line (4T1) were procured from ATCC. Cultures of these cell lines were sustained in DMEM supplemented with 10% FBS and 1% PSN. Patient-derived cells (TX-BR-109, TX-BR-237, TX-BR-247, TX-BR-313, and TX-BR-290) were procured from the Children’s Oncology Group (Texas Tech University Science Center, Lubbock, TX, USA) and cultured in DMEM supplemented with 20% FBS, 1% PSN, and 1× ITS (5 µg/mL insulin, 5 µg/mL transferrin, 5 µg/mL selenous acid). Authentication of all mentioned cell lines was routinely conducted through short tandem repeat (STR) analysis.

### 2.3. Sulforhodamine B Assay (SRB)

A cytotoxicity assay was performed using SRB dye as described by us previously [31]. Briefly, 4000–5000 cells/well were plated in 96-well plates. The following day, a wide range of proguanil (0–100 μM) was added to the cells. Following incubation intervals of 24, 48, and 72 h, cell fixation was performed using 10% trichloroacetic acid, followed by staining with SRB dye. Subsequently, optical density measurements were taken with a 10 mM Tris base solution.

### 2.4. Proliferation Assay

Carboxyfluorescein succinimidyl ester (CFSE) was obtained and prepared as per the manufacturer’s instruction (Invitrogen Cat. No. C34571 (Waltham, MA, USA)). Cells were incubated with 5µM CFSE for 20 min at 37 °C. Free dye was removed by adding five volumes of culture media to the cells, and the cells were seeded into 6-well plates. After 24 h, cells were treated with proguanil or vehicle (DMSO control) for a specified time, stained with AmCyan, and analyzed using a flow cytometer.

### 2.5. Annexin V-FITC Apoptosis Assay

The apoptosis assay was conducted following our previously described protocol using annexin-V/FITC and propidium iodide [32]. In summary, approximately 0.2 × 10^6^ cells were seeded in each well of a six-well plate and subjected to varying concentrations of proguanil for 72 h. The Annexin/PI kit was employed for the apoptosis assay, and the results were analyzed using a BD flow cytometer.

### 2.6. Generation of Reactive Oxygen Species

Intracellular ROS levels were assessed by quantifying the production of superoxide and hydrogen peroxide ions in cells using dihydroethidium (DHE) and 6-carboxy-2, 7-dichlorodihydrofluorescein diacetate (DCFDA) dyes through flow cytometry. Briefly, 0.2 × 10^6^ cells were seeded and allowed to adhere overnight in each well of six-well plates. Subsequently, the cells were treated with either DMSO or 40 and 60 μM proguanil for varying durations. Following exposure, cells were incubated with 10 μM DCFDA and 5 μM hydroethidine at 37 °C for 25 min. Post-incubation, cells were extracted, washed, and resuspended in PBS for analysis of ROS generation using a BD flow cytometer. Each sample was evaluated for approximately 10,000 cells, excluding cell debris and clumps from the analysis.

In order to block ROS generation, NAC or mitochondria-specific MitoTempo was used. Briefly, 4000–5000 cells/well were plated in 96-well plates. Cells were pretreated with 5 mM NAC (Sigma #138061) [33] or 2 µM Mito Tempo (MedChem Express #HY-112879 (Monmouth Junction, NJ, USA)) [34] for 1 h before treatment with proguanil. A cytotoxicity assay was performed using SRB dye as described previously.

### 2.7. Fluorescence Microscopy

Around 0.2 × 10^6^ cells were plated in each well of 6-well plates containing poly-L-Lysine-coated coverslips. Cells were allowed to attach overnight. The next day, the coverslips were rinsed twice with PBS and co-stained with MitoTracker Green (100 nM in HBSS) and MitoSOX Red (5 µM in HBSS) as specified by the manufacturer (ThermoFisher #M36008 and #M7514 (Waltham, MA, USA)). Following the staining, cells were treated with media containing DMSO or proguanil for 15 min, rinsed with PBS, and fixed using 4% paraformaldehyde (PFA). Fluorescence images from a total of 100 cells per coverslip were obtained using a Nikon Eclipse TE2000-E confocal microscope and analyzed using NIS-elements AR analysis 4.60.00 64-bit and GraphPad Prism 9 software.

### 2.8. Mitochondrial Membrane Potential

Changes in mitochondrial membrane potential were assessed through flow cytometry employing the membrane-potential-sensitive dye tetramethyl rhodamine (TMRM), which is taken up by active mitochondria with intact membrane potentials. Briefly, control and proguanil-treated cells after the desired duration of treatment were incubated with 10 μM TMRM at 37 °C for 15 min, harvested, washed, and resuspended in cold PBS. Cells were further analyzed using a flow cytometer.

### 2.9. Seahorse XFe-24 Metabolic Flux Analysis

Real-time determination of oxygen consumption rates (OCRs) in HCC1806 and MDA-MB-231 cells was conducted using the Seahorse Extracellular Flux (XFe-24) analyzer (Seahorse Bioscience, Chicopee, MA, USA) following the provided protocol by Seahorse Bioscience. Each well of XFe-24-well cell culture plates was seeded with approximately 30,000–40,000 cells and incubated overnight for attachment. Subsequent to seeding, cells were exposed to proguanil (20 μM and 40 μM) for 48 h, with parallel processing of vehicle-only (DMSO) control cells. Post-treatment, cells were rinsed in XF assay media supplemented with 1 mM pyruvate, 10 mM glucose, and 2 mM L-glutamine, adjusted to a pH of 7.4. Cells were then incubated for 1 h in 500 μL/well of XF assay media at 37 °C in a non-CO_2_ incubator, followed by immediate analysis using a Flux analyzer. Data analysis was performed using XFe-24 software (v 3.0.11).

### 2.10. Western Blot Analysis

Breast cancer cells underwent treatment with varying concentrations of proguanil for a duration of 72 h. Whole-cell lysates were prepared using 4% (*w/v*) CHAPS buffer, while RIPA lysis buffer was employed to lyse tumor samples post-homogenization in 1× PBS. Protein quantification was achieved using a Bradford reagent. Subsequently, SDS-PAGE was conducted with 40–60 μg of protein, and the separated proteins were transferred onto a PVDF membrane. The membranes were probed for primary antibodies against p-H2AX#7631, Bax #2774S, Bcl-2 #3498S, survivin #2808, C-caspase-9 #7237S, C-caspase-3 # 9661S, C-PARP #5625, and β-actin #SAB5600204. Except for β-actin, which was purchased from Sigma Aldrich (St Louis, MO, USA), all primary antibodies were obtained from Cell Signaling Technologies (Danvers, MA, USA). The membrane was developed as described by us previously [35]. The protein levels that correspond to immunoreactive bands were quantified using ImageJ v1.53e image analysis software (National Institutes of Health (Bethesda, MD, USA)), with normalization against beta actin as an internal loading control.

### 2.11. In Vivo Tumor Model

Female Balb/c mice (4–6 weeks old) were procured from Envigo (Indianapolis, Indiana). All animal experiments adhered to ethical standards and were conducted following protocols approved by the Institutional Animal Care and Use Committee (IACUC). In brief, orthotopic injections of 0.07 × 106 4T1 tumor cells in 0.1 mL PBS were administered to the left and right 3rd mammary fat pad. Once the tumor size reached approximately 80–100 mm^3^, mice were randomly assigned to two groups, each comprising 5 mice. Given that each mouse bore two tumors, each group encompassed a total of 10 tumors. Treatment involved daily administration of 20 mg/kg proguanil starting day 5 after tumor cell implantation and continued for a period of 27 days.

### 2.12. Statistical Analysis

Analysis and graphical presentations of data were conducted using GraphPad Prism (Version 7.0). Results are expressed as means ± SD or SEM derived from a minimum of three independent experiments, unless otherwise indicated. To assess the statistical significance of differences between control and treated groups, the Student’s *t*-test and Mann–Whitney test were employed for parametric and non-parametric data, respectively. P-values below 0.05 were regarded as statistically significant.

## 3. Results

### 3.1. Proguanil Has Anti-Proliferative Effects on Breast Cancer Cells

**Figure 1:**
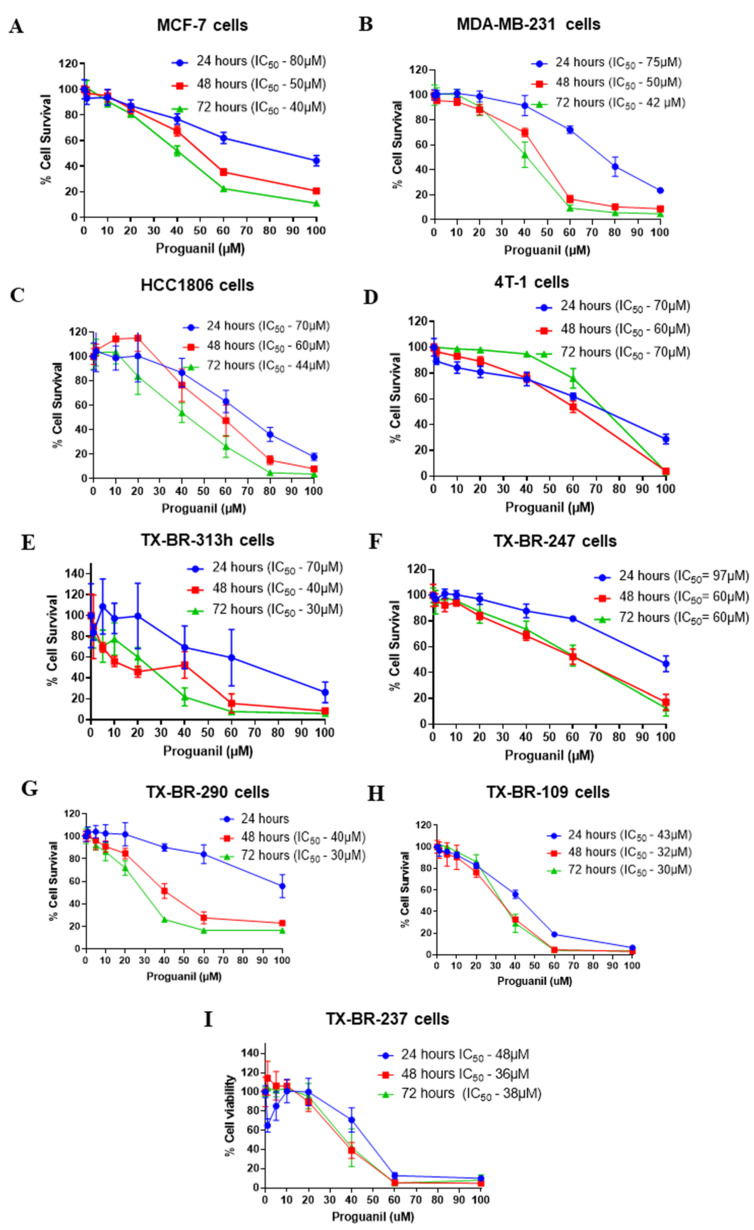
Anti-proliferative effects of proguanil in breast cancer cell lines and patient-derived breast cancer cells. (**A**) MCF-7, (**B**) MDA-MB-231, (**C**) HCC1806, (**D**) 4T1, (**E**) TX-BR-313 h, (**F**) TX-BR-247, (**G**) TX-BR-290, (**H**) TX-BR-109, and (**I**) TX-BR-237. The cells were treated with increasing concentrations of proguanil for 24, 48, and 72 h. Cell proliferation was measured by Sulforhodamine B assay. The experiments were repeated at least three times with eight replicates in each experiment.

To evaluate the anti-proliferative activity of proguanil, we first investigated the effects of proguanil on the growth of breast cancer cells. We used a panel of breast cancer cells including human, murine, and several patient-derived cell lines. Proguanil treatment significantly reduced the proliferation of all the cell lines in a dose- and time-dependent manner with IC_50_ in the range of 40–70 µM (Figure 1A–I). It is worth noting that proguanil was more effective in suppressing the growth of patient-derived breast cancer cell lines (Figure 1). The IC_50_ of proguanil after 72 h treatment ranged from 30 to 60 µM in all five patient-derived cell lines (Figure 1E–I). These data suggested the potential anti-proliferative effects of proguanil in breast tumor cells.

**Figure 2:**
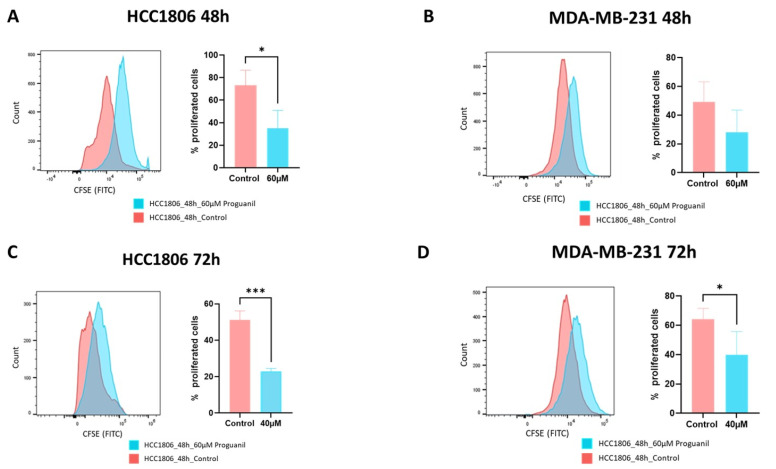
Inhibition of cell proliferation by proguanil. After staining with CFSE, (**A**,**C**) HCC1806 cells and (**B**,**D**) MDA−MB−231 cells were treated with proguanil for 48 and 72 h and then analyzed by flow cytometer. Each experiment was performed in triplicate (*n* = 3). Parametric one-tailed student *t*-test was used for statistical analysis. Statistically significant compared with control: * *p* < 0.05; *** *p* < 0.001.

To confirm the anti-proliferative effects of proguanil, we stained the cells with carboxyfluorescein succinimidyl ester dye (CFSE). CFSE is a widely used tool for tracking cell division. CFSE attaches covalently to intracellular compounds using the fluorescent dye, carboxyfluorescein. Consequently, as a CFSE-labeled cell splits, its offspring possess a reduced quantity of carboxyfluorescein-labeled compounds. This allows for the evaluation of each division by gauging the corresponding decline in cellular fluorescence via flow cytometry. Our results showed that when the cells were treated with proguanil, there was no shift in CFSE fluorescence, indicating there was no or little cell division compared to the control (Figure 2). These results confirmed the anti-proliferative effects of proguanil on breast cancer cells.

### 3.2. Proguanil Triggers Apoptosis in Breast Cancer Cells

**Figure 3:**
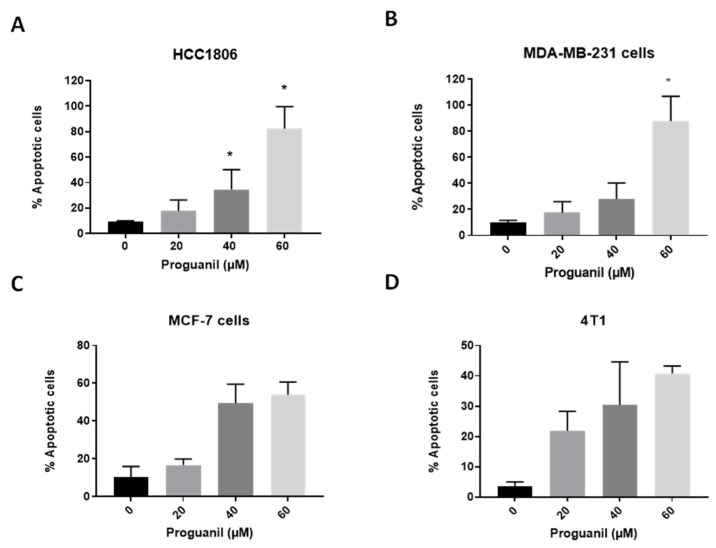
Apoptosis-inducing effects of proguanil in breast cancer cell lines. (**A**) HCC1806, (**B**) MDA-MB-231, (**C**) MCF-7, and (**D**) 4T1 cells were treated with 20–60 μM proguanil for 72 h. Apoptotic cells were determined by Annexin V/PI assay using a flow cytometer. Each experiment was performed in triplicate (*n* = 3). Non-parametric student *t*-test (Mann-Whitney test) was used for statistical analysis. Statistically significant when compared with control, * *p* < 0.05.

We further determined the mechanism of proguanil’s inhibitory effect in different breast cancer cell lines. HCC1806, MDA-MB-231, MCF-7, and 4T1 cells were treated with various concentrations of proguanil for 72 h and analyzed for apoptosis using the Annexin V/PI assay. We observed a time- and concentration-dependent increase in the apoptotic cells after proguanil treatment in all cell lines (Figure 3A–D). Treatment with 60 µM proguanil for 72 h resulted in more than 80% apoptotic cell death of HCC1806 and MDA-MB-231 cells. On the other hand, MCF-7 and 4T1 were less sensitive to proguanil treatment. A potential reason could be the difference in the origin of the cells (human vs. murine) as well as the difference in the cell types (basal vs. luminal).

### 3.3. Proguanil Causes the Generation of Mitochondrial ROS in Breast Cancer Cells

To further dissect the mechanism of proguanil-induced apoptosis, we sought to determine if proguanil has any effect on ROS generation in breast cancer cell lines. Intracellular ROS generation by proguanil was evaluated by flow cytometry using 2’,7’-dichlorofluorescin diacetate (DCFDA) and hydroethidine (DHE). DHE is a fluorogenic dye that can freely permeate cell membranes and be oxidized by cellular O2^•−^. Following oxidation, it produces two red-fluorescent products: (a) ethidium (E+) and (b) 2-hydroxyethidium (2-OH-E+). Among these two, 2-hydroxyethidium is a specific adduct of cellular O2^•^. Another fluorogenic dye, DCFDA, assesses peroxyl, hydroxyl, and other reactive oxygen species (ROS) activity within the cell. DCFDA permeates the cell and undergoes deacetylation by cellular esterases, transforming into a non-fluorescent compound. This non-fluorescent compound is subsequently oxidized by ROS to produce 2′, 7′–dichlorofluorescein (DCF). The highly fluorescent nature of DCF allows for detection using fluorescence spectroscopy.

**Figure 4:**
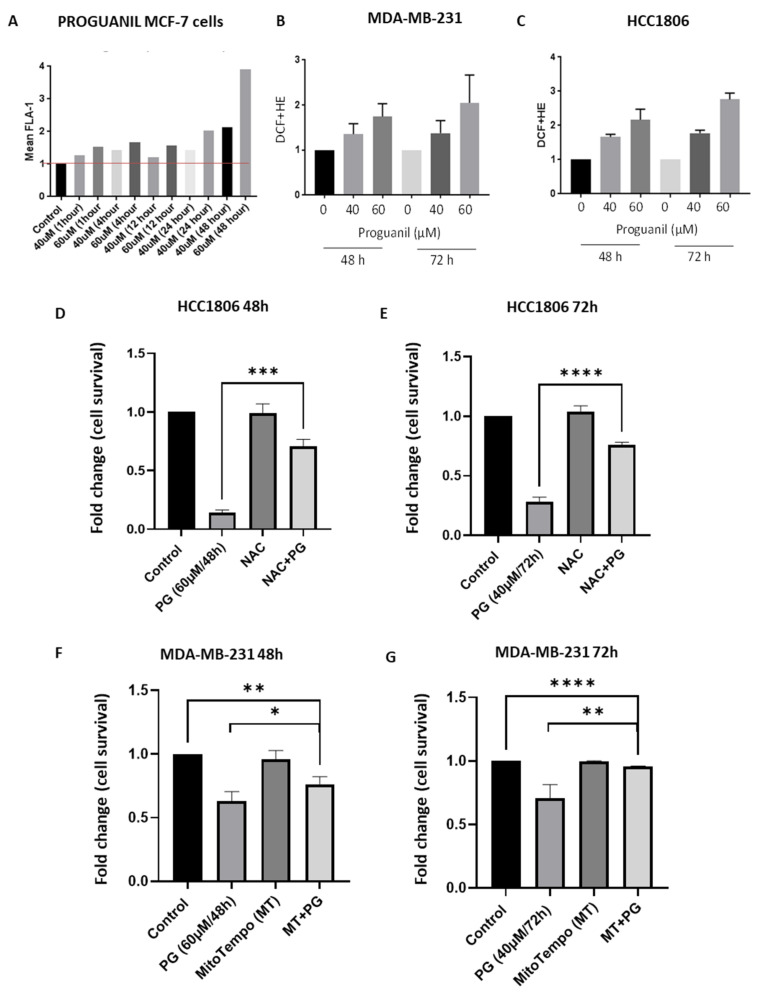
Effect of proguanil on the generation of reactive oxygen species (ROS). (**A**) A time-dependent study of ROS in MCF-7 cell line. Cells were treated with 40µM and 60µM proguanil for various time points and processed as described in the Method section. (**B**) Effects of proguanil on the generation of ROS in MDA-MB-231 and (**C**) HCC1806. The cells were treated with proguanil for 48 or 72 h and analyzed for DCF and HE fluorescence (ROS generation) by flow cytometer upon staining the cells with DCFDA and DHE. (**D**,**E**) Antioxidant N-acetyl cysteine (NAC) protects HCC1806 cell viability by blocking ROS generation induced by proguanil. HCC1806 cells were pretreated with 5 mM NAC for 3 h or 2 µM MitoTemp for 1 h followed by treatment with (**D**,**F**) 60 µM proguanil for 48 h or (**E**,**G**) 40 µM proguanil (PG) for 72 h, and the viability of cells was determined by SRB assay. Results are shown as the mean ± SD (*n* = 3). Non-parametric student *t*-test (Mann-Whitney test) was used in (**A**–**C**), while parametric one-tailed student *t*-test was used for statistical analysis in (**D**–**G**) Statistically significant: * *p* < 0.05; ** *p* < 0.01; *** *p* < 0.001; **** *p* < 0.0001.

A pilot time-dependent study of ROS generation was conducted initially in MCF-7 cells to determine the optimum time for ROS generation. Maximum ROS generation was observed at 48 h by proguanil; 48 and 72 h time points were used for further studies (Figure 4A). As shown in Figure 4B,C, exposure of breast cancer cells to proguanil for 48 h and 72 h led to a dose-dependent elevation in DCF and HE fluorescence, representing total reactive oxygen species (ROS) generation. In comparison to the DMSO-treated control, the increase in ROS generation due to proguanil treatment was amplified 1.9- and 2.1-fold at 72 h in MDA-MB-231 and HCC1806 cells, respectively. This observation signifies that proguanil triggers the generation of ROS in breast cancer cells.

In order to assess the role of ROS in proguanil-induced cytotoxicity, HCC1806 cells were treated with N-Acetylcysteine (NAC), an antioxidant, prior to treatment with proguanil. NAC is known for reducing oxidative stress by scavenging ROS. Our results indicate that treatment with NAC blocked the effects of ROS induced by proguanil (Figure 4D,E). The reduction in the viability of HCC1806 cells by proguanil was significantly prevented by NAC treatment, indicating the role of ROS in the anti-proliferative effects of proguanil (Figure 4D,E).

To confirm the role of mitochondria in the generation of ROS by proguanil, we used the mitochondria-specific antioxidant MitoTempo (MT). MT is a conjugate of the antioxidant Tempol with the lipophilic triphenylphosphonium cation, TPP+ [36]. Tempo functions as a SOD mimetic, dismutating superoxide during the catalytic cycle, while TPP+ is a cation permeable to membranes, accumulating significantly within mitochondria due to the membrane potential [36]. This fusion produces a mitochondria-targeted compound with potent superoxide scavenging abilities. The aim of our experiments was to assess whether MT could shield against proguanil-induced cytotoxicity by mitigating mitochondrial ROS/oxidative stress and dysfunction. Our results show that MitoTempo significantly mitigated the cytotoxic effects of proguanil, indicating the role of mitochondrial ROS in the cytotoxic effects of proguanil (Figure 4F,G).

To further confirm that ROS production originated in the mitochondria, we analyzed proguanil-treated HCC1806 cells using a fluorescence microscope. The cells were co-stained with MitoSOX Red and MitoTracker Green fluorogenic dyes and fixed with 4% PFA. MitoTracker Green is a green-fluorescent mitochondrial stain that localizes in the mitochondria of living cells irrespective of the mitochondrial membrane potential. MitoSOX™ Red dye permeates live cells and then selectively targets mitochondria. Inside mitochondria, MitoSOX™ Red is rapidly oxidized by superoxide radicals.

**Figure 5:**
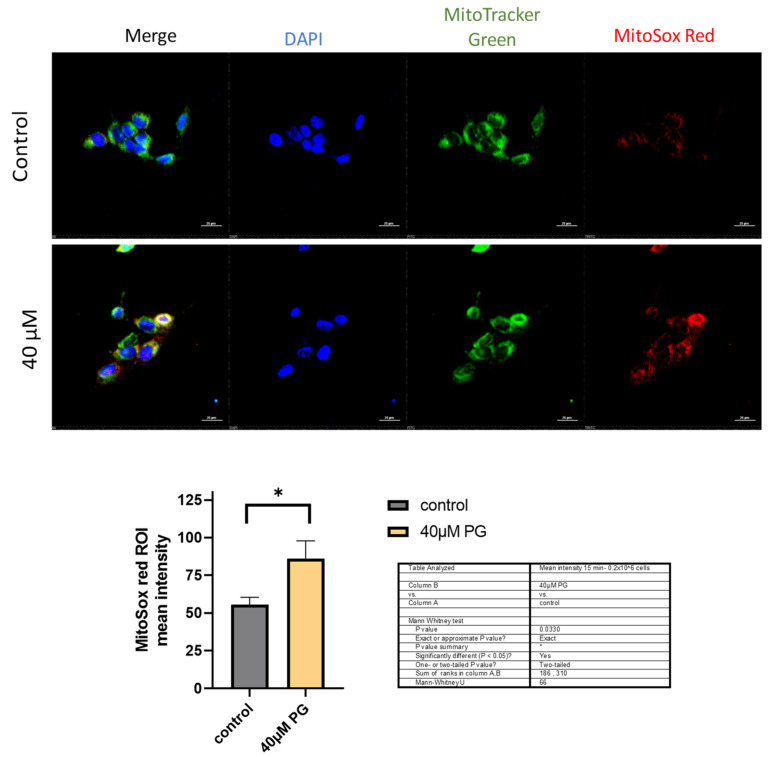
Proguanil induces increased superoxide production in mitochondria. Fluorescence microscopy on HCC1806 cells performed using MitoTracker Green and MitoSOX Red stains. Fluorescence images from about 100 cells per coverslip were obtained using Nikon Eclipse TE2000-E confocal microscope and analyzed using NIS-elements AR analysis 4.60.00 64-bit and GraphPad Prism 9 software. Non-parametric student *t*-test (Mann-Whitney test) was used for statistical analysis. * Statistically significant when compared with control, *p* < 0.05.

Our results showed a significant increase in MitoSOX Red fluorescence as early as 15 min post-treatment with proguanil (Figure 5). The overlap of the two dyes confirms that proguanil induces the generation of superoxide radicals specifically in the mitochondria of breast cancer cells.

### 3.4. Proguanil Disrupts Mitochondrial Membrane Potential in MDA-MB-231 and HCC1806 Cells

**Figure 6:**
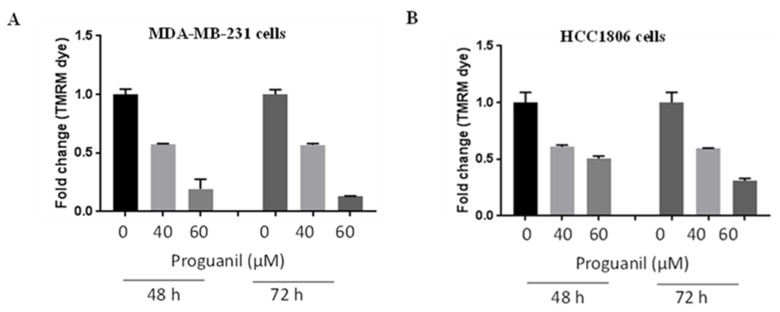
Effect of proguanil on mitochondrial membrane potential. Fold change with TMRM fluorescence in (**A**) MDA-MB-231 and (**B**) HCC1806 cell cultures treated with 40 or 60 μM proguanil for the indicated time periods. Data are mean ± S.E. (*n* = 3). Non-parametric student *t*-test (Mann-Whitney test) was used for statistical analysis.

Increased intracellular ROS leads to apoptotic cell death by disrupting mitochondrial membrane potential. We used tetramethyl rhodamine, methyl ester (TMRM), which permeates the cell membrane and then accumulates in active mitochondria with intact membrane potentials. As the mitochondrial membrane potential is lost, TMRM accumulation decreases. The disruptions in ΔΨm by proguanil were measured by staining the cells with TMRM and then analyzed by flow cytometry. As shown in Figure 6A,B, proguanil treatment significantly reduces the accumulation of TMRM dye in MDA-MB-231 and HCC1806 cells, in a concentration-dependent manner, when compared with control. This indicates the reduction in mitochondrial membrane potential by proguanil in breast cancer cells.

### 3.5. Proguanil Treatment Activates the Caspase-3 Cascade in Breast Cancer Cells

**Figure 7:**
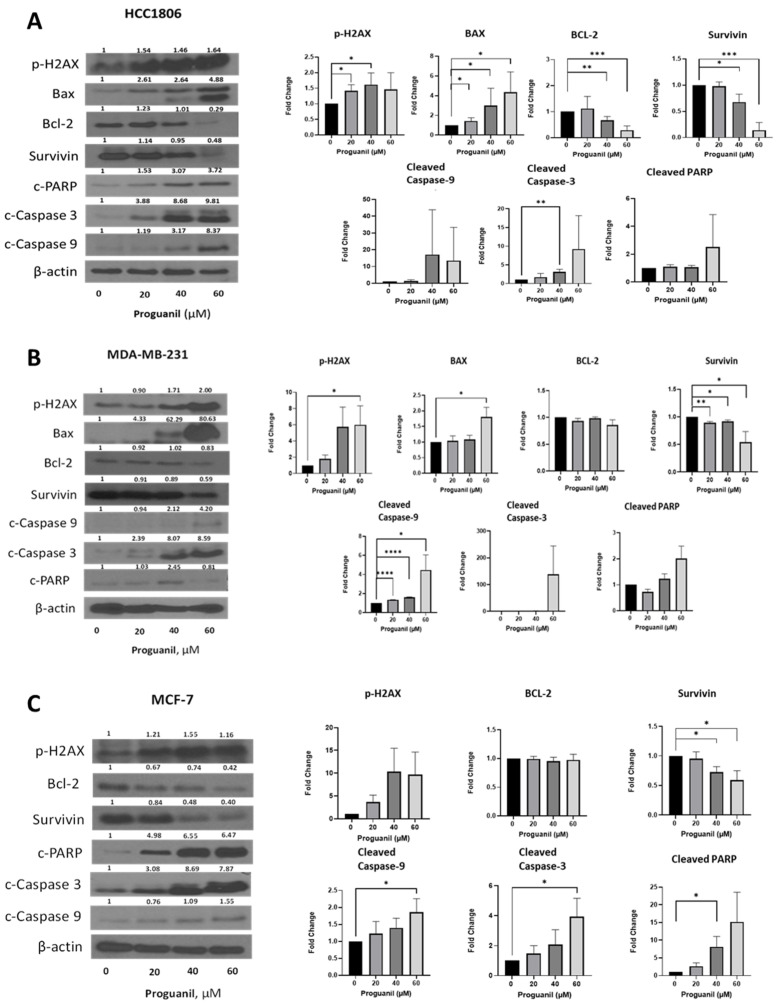
Proguanil activates the intrinsic cell death pathway. Western blot results and corresponding statistical analysis for (**A**) HCC1806, (**B**) MDA-MB-231, and (**C**) MCF-7. The cells were treated with 20–60 μM proguanil for 72 h. Western blot analyses showing the immunoblots for p-H2AX, bax, bcl-2, survivin, cleaved fragments of caspase-9 and caspase-3, and PARP using appropriate antibodies. Blots were stripped and re-probed for actin to ensure equal protein loading. These experiments were performed 3–5 times independently, with similar results obtained in each experiment. Statistical significance was evaluated using parametric one-tailed student *t*-test and compared with control: * *p* < 0.05; ** *p* < 0.01; *** *p* < 0.001; **** *p* < 0.0001. Original full western blots are available in File S1.

To evaluate the modulation of key signaling molecules of the mitochondrial death pathway after proguanil treatment, we performed Western blot analyses in the lysates of HCC1806, MDA-MB-231, and MCF-7 cells treated with different concentrations of proguanil. Our results revealed that proguanil treatment for 72 h in HCC1806 cells resulted in increased expression of p-H2AX, which is a DNA damage marker (Figure 7A). In addition, we also observed increased expression of Bax, an apoptosis marker, and decreased expression of Bcl-2 and survivin, which are involved in apoptosis inhibition, in response to proguanil treatment (Figure 7A). Moreover, proguanil treatment caused significant activation of caspase-3, caspase-9, and PARP as evidenced by their cleavage (Figure 7A). Similar results were obtained in MCF-7 and MDA-MB-231 cells following exposure to proguanil for 72 h (Figure 7B–C). Notably, as the concentration of proguanil increased, the ratio of Bax to Bcl-2 also increased, indicating an increase in apoptosis. Collectively, these results suggest that proguanil activates the intrinsic mitochondrial death pathway in breast cancer cells.

### 3.6. Proguanil Inhibits Oxidative Phosphorylation of Breast Cancer Cells

**Figure 8:**
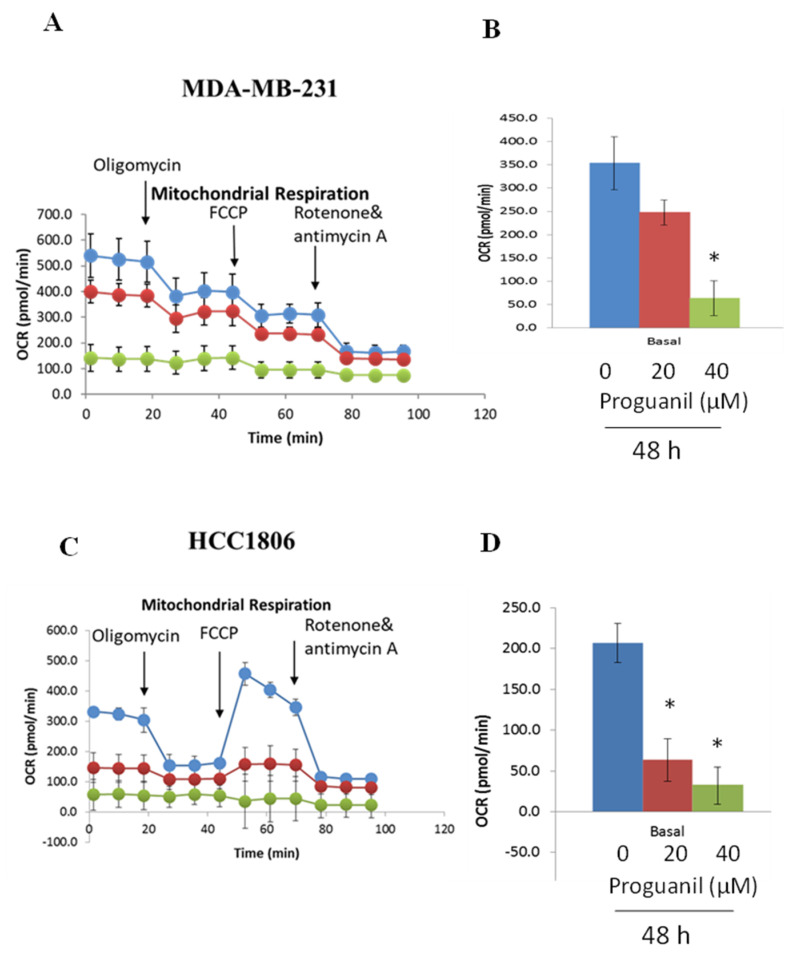
Proguanil treatment inhibits the mitochondrial respiration of MDA−MB−231 (**A**,**B**) and HCC1806 (**C**,**D**) breast cancer cells. The metabolic profile of breast cancer cell monolayers treated with proguanil (Control—blue; 20 μM—red; 40 μM—green) was assessed using the Seahorse XF-e24 analyzer. The oxygen consumption rates of control and proguanil-treated cells were significantly different as per Non-parametric student *t*-test (Mann-Whitney test) (* *p* < 0.05).

Given that proguanil activates the mitochondrial death pathway after ROS generation and disruption of the mitochondrial membrane, the next step was to examine proguanil’s effect on the mitochondrial respiration of cancer cells. Utilizing the Seahorse XF-e24 analyzer, we evaluated the metabolic profile of HCC1806 and MDA-MB-231 cells treated with increasing concentrations of proguanil at 48 h. Our findings indicate a substantial concentration-dependent inhibition of the oxygen consumption rate (OCR) in both MDA-MB-231 and HCC1806 cell lines due to proguanil treatment (Figure 8A–D). These results strongly imply that proguanil hampers the oxidative phosphorylation process in breast cancer cells.

### 3.7. Proguanil Inhibits the Growth of Orthotopically Implanted 4T1 Breast Tumors

**Figure 9:**
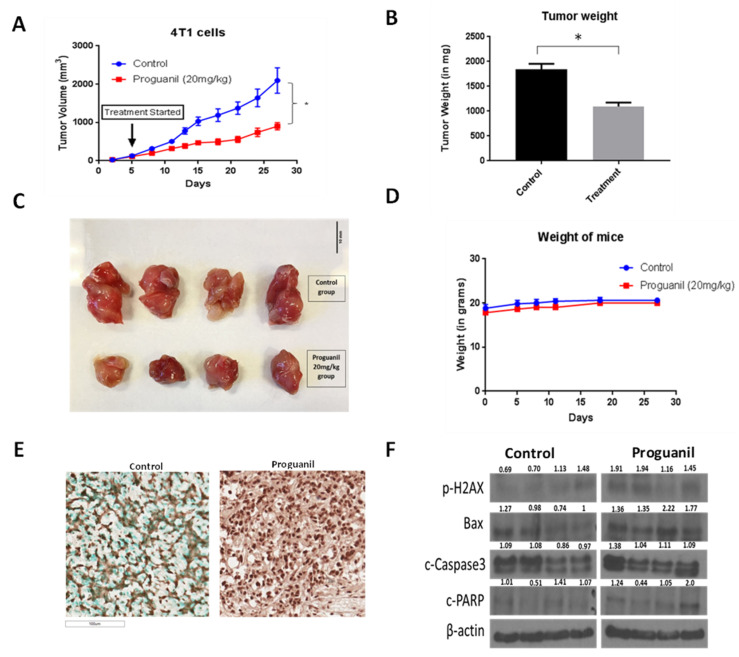
In vivo antitumor activity of proguanil in an orthotopic breast cancer model. (**A**) Tumor growth curve of 4T1 tumors from control and proguanil-treated mice. Values are plotted as mean ± SEM. (**B**) Average tumor weight obtained from control and proguanil-treated mice. (**C**) Tumor images of control and proguanil-treated mice. (**D**) Weight of the mice during the study. (**E**) TUNEL assay in the tumors of control and proguanil-treated mice. (**F**) Western blots of the tumors of vehicle- and proguanil-treated mice. 4T1 tumors were minced, lysed, and analyzed for p-H2AX, Bax, c-caspase-3, and c-PARP. Each band represents a tumor from an individual mouse. Statistically significantly different compared with control as analyzed by parametric student’s *t*-test (* *p* < 0.05). Original full western blots are available in File S1.

Thus far, we observed that proguanil was able to suppress the growth of various breast cancer cells by inducing apoptosis and ROS generation. To evaluate the efficacy of proguanil in vivo, a highly aggressive 4T1 murine breast cancer cell line was used. 4T1 cells were implanted in the third mammary fat pad of female Balb/c mice, and once the tumor was established, mice were administered 20 mg/kg proguanil daily by oral gavage. Our results showed that the growth rate of 4T1 tumors was significantly inhibited after proguanil treatment as compared to the vehicle-treated mice (Figure 9A–C). On the day of termination, tumors from the control and proguanil-treated mice were excised and weighed. The average net weight of the tumors from proguanil-treated mice was significantly less when compared with the control group (Figure 9B,C). The average body weight of control and proguanil-treated mice did not change throughout the study, suggesting no obvious toxicity at this dose (Figure 9D). Western blot analyses from the control and treated tumors showed that proguanil-treated tumors exhibited increased levels of p-H2AX, Bax, c-PARP, and c-caspase3 (Figure 9F). Additionally, TUNEL assay results further confirmed the pro-apoptotic effect of proguanil in the in vivo tumor samples (Figure 9E).

## 4. Discussion

Proguanil and other biguanide compounds are positively charged molecules that accumulate in the mitochondrial matrix at concentrations that are 1000-fold higher than extracellular concentrations. The concentration depends on the presence of transporters and also the cell and mitochondrial membrane potential [25]. There is little information about the transport of proguanil into the mitochondria, but in yeast and malaria parasites, proguanil most likely accesses the mitochondria. Its synergy with atovaquone, which specifically targets the bc_1_ complex, indicates mitochondria to be the location of proguanil’s activity [37].

There is now considerable evidence supporting the antineoplastic role of anti-parasitic drugs [25,31,38,39,40,41], but only a few studies have recognized the cytotoxic effects of proguanil and attempted to delineate its mechanism of action [23,27,28,42]. Some studies suggested that this drug is unable to access the mitochondrial matrix of mammalian cells, although it is an effective inhibitor of isolated Complex I [24,29,37]. In contrast, other studies suggest that proguanil may exert anticancer effects by reducing hypoxia, inducing oxidative stress and mitochondrial dysfunction, and causing DNA damage [26].

Our results showed that proguanil exerts strong anti-proliferative effects not only in established breast cancer cell lines but also in several patient-derived cell lines. Our results further indicate that the antineoplastic effects of proguanil were associated with the increased production of ROS in the mitochondria, disruption of oxidative phosphorylation, and consequentially DNA damage and apoptosis.

Mitochondria have been recognized as the main intracellular source of ROS. Under normal physiological conditions, for mitochondria that are actively producing ATP, the rate of ROS generation is lower. This is also due to the adequate levels of antioxidants that avert ROS accumulation and oxidative damage [42]. Several studies reported that ROS play an important role in the initiation and progression of cancer [9,43,44,45,46,47,48,49]. On the other hand, numerous studies have demonstrated that enhanced ROS can suppress tumor growth [10,12,13,14,50,51,52,53]. This implies that cancer cells evolved by fine-tuning levels of ROS and their redox environments. Thus, when this fine balance is disrupted, cancer cells can be destroyed by the same mechanism that aids their survival.

The dual effect of ROS is also evident in breast cancer. Many chemotherapeutic and radiotherapeutic agents used for breast cancer treatment work by augmenting ROS and oxidative stress in cells [15,50]. Therefore, targeting ROS homeostasis in breast cancer has been proven as an effective strategy. Our results indicated that proguanil caused about 2-3-fold increased ROS generation in HCC1806 and MDA-MB-231 cells compared to the control. In contrast to other studies showing that proguanil does not inhibit mitochondrial respiration, our results showed a significant reduction in the OCR and ATP production rate after proguanil treatment. Fluorescence microscopy results confirmed the mitochondrial origin of ROS as well as the early generation of O_2_^•^ after proguanil treatment. The divergence in findings between our study and other studies could be due to the differences in the proguanil concentrations and the duration of the treatment [45].

Our study showed that proguanil exerts potent anticancer activity in vivo as well. Size reductions of animal breast tumors and an increase in apoptotic markers were observed after oral administration of 20 mg proguanil/kg every day. 4T1 tumors are highly aggressive and metastatic and represent stage IV human breast cancer [26]. Proguanil treatment suppressed 4T1 breast tumor growth by 55% in female Balb/c mice in our study. The tumors from proguanil-treated mice showed increased apoptosis, which was associated with the increased expression of Bax and p-H2AX.

Proguanil is given at a dose ranging from 100 to 400 mg for the treatment of malaria in humans. The dose of proguanil used in our study was 20 mg/kg in mice, which, when converted to a human equivalent dose, is 1.66 mg/kg. Thus, a human equivalent dose of proguanil would be approximately 96 mg for a person with a body weight of 60 kg, which is less than the lowest dose given to humans for the treatment of malaria, alleviating any concerns related to the dose or potential side effects in humans.

## 5. Conclusions

The current study provides evidence that proguanil induces apoptosis in breast cancer cells through the activation of the mitochondrial death pathway. While the specific mechanism by which proguanil achieves this effect still remains to be fully understood, the results suggest that it may be related to the generation of reactive oxygen species (ROS). Nevertheless, oral administration of proguanil remarkably suppresses the growth of breast tumors by inducing apoptosis in the tumor cells.

In conclusion, the present study provides compelling evidence justifying that proguanil is an effective in vitro and in vivo inhibitor of breast cancer and hence should be further considered for clinical investigation against breast cancer.

## Data Availability

Data are contained within the article and supplementary materials.

## Supplementary Materials

The following supporting information can be downloaded at: https://www.mdpi.com/article/10.3390/cancers16050872/s1, File S1: Original western blots.

## List of Abbreviations

4T1	murine breast cancer cell line
ATCC	American Type Culture Collection
Bax	Bcl-2-associated X protein
bcl-2	B-cell lymphoma 2 protein
Balb/c	Balb/c mice
CFSE	carboxyfluorescein succinimidyl ester dye
DCFDA	6-carboxy-2, 7-dichlorodihydrofluorescein diacetate
DHE	dihydroethidium
DMEM	Dulbecco’s Modified Eagle Medium
FBS	fetal bovine serum
HCC1806	human breast carcinoma cell line
ITS	insulin–transferrin–selenous acid
MCF-7	human breast carcinoma cell line
MDA-MB-231	human breast carcinoma cell line
PSN	penicillin–streptomycin–neomycin
SRB	Sulforhodamine B assay
STR	short tandem repeat
TMRM	tetramethyl rhodamine
TX-BR	patient-derived cells
ROS	reactive oxygen species
