# Supplementary material for: Proguanil Suppresses Breast Tumor Growth In Vitro and In Vivo by Inducing Apoptosis via Mitochondrial Dysfunction
Source: Cancers (Basel). 2024 Feb 22;16(5):872. doi: 10.3390/cancers16050872 (PMC10931526; doi:10.3390/cancers16050872)

Western blot raw data
Figure 7

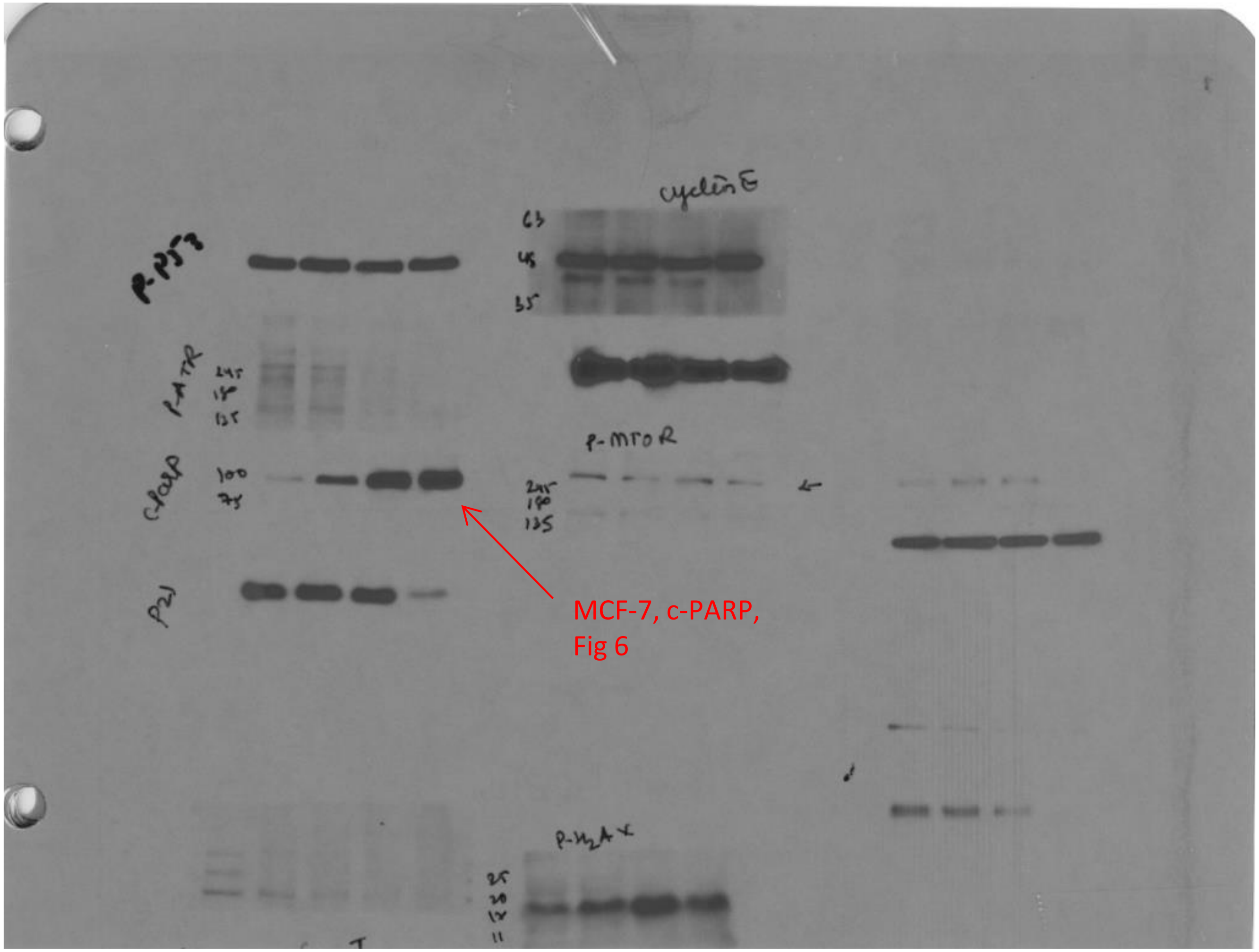

MCF-7, c-PARP,
Fig 6

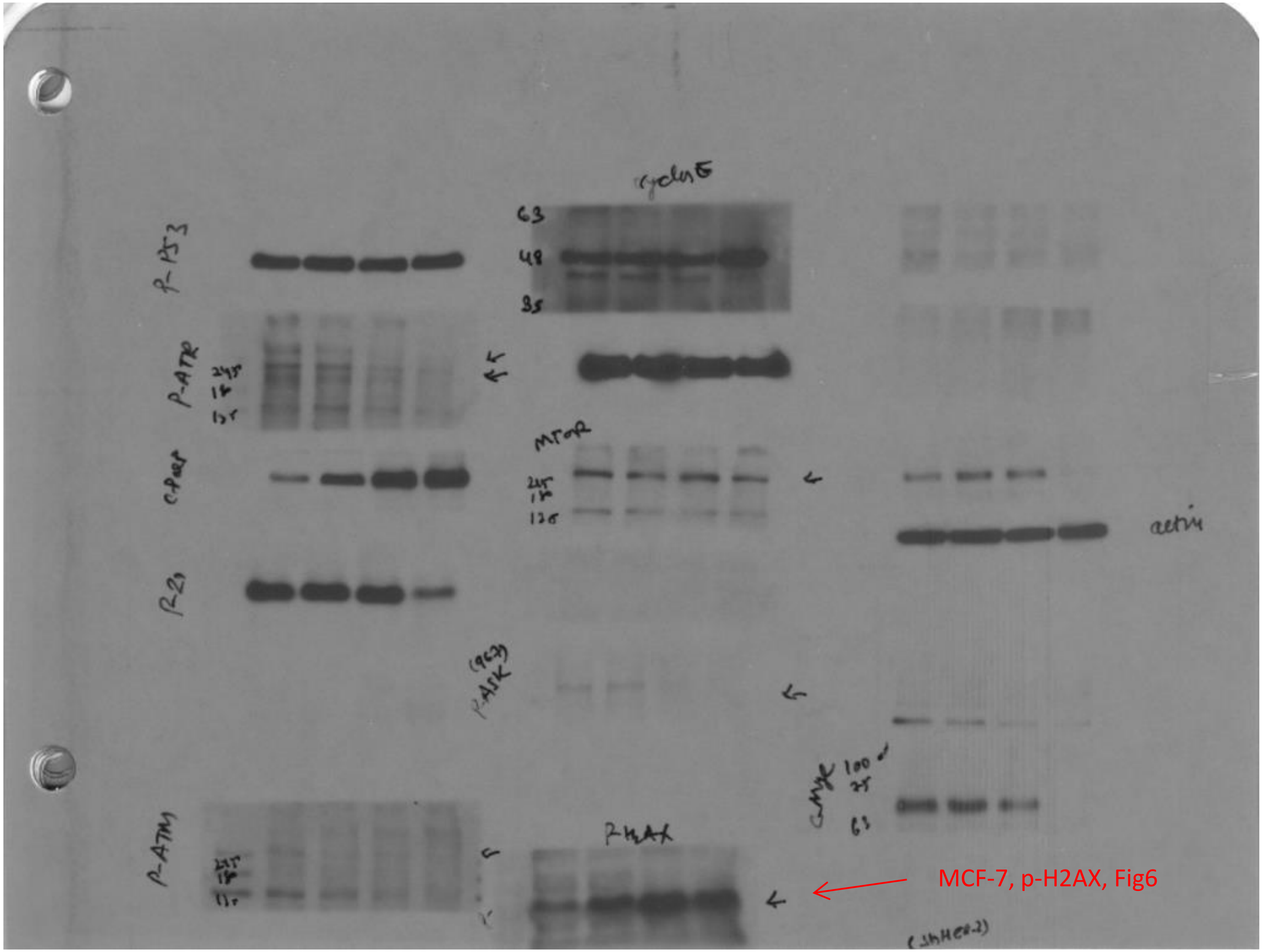

MCF-7, p-H2AX, Fig6

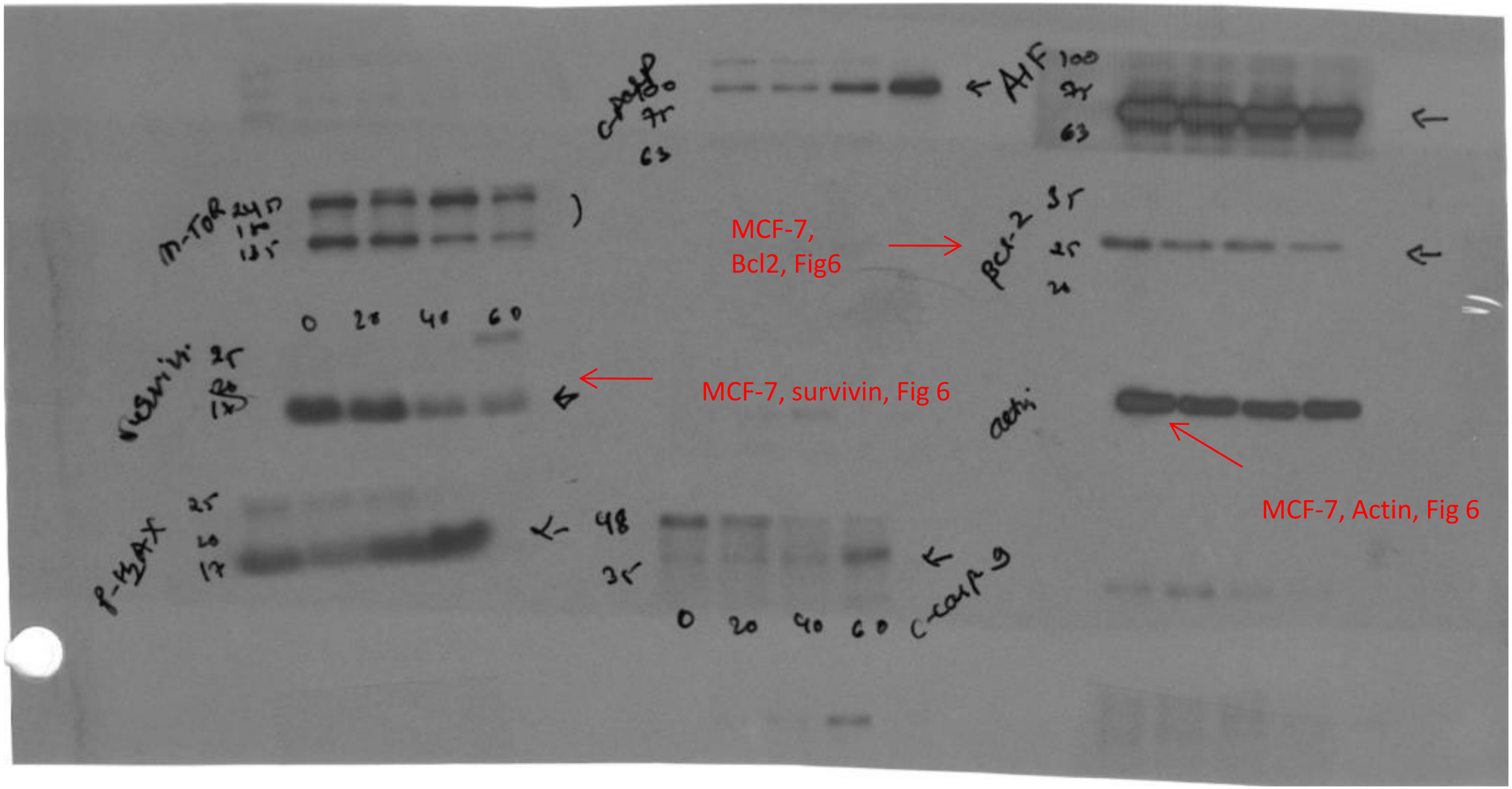
MCF-7,
Bcl2, Fig6
MCF-7, survivin, Fig 6
MCF-7, Actin, Fig 6

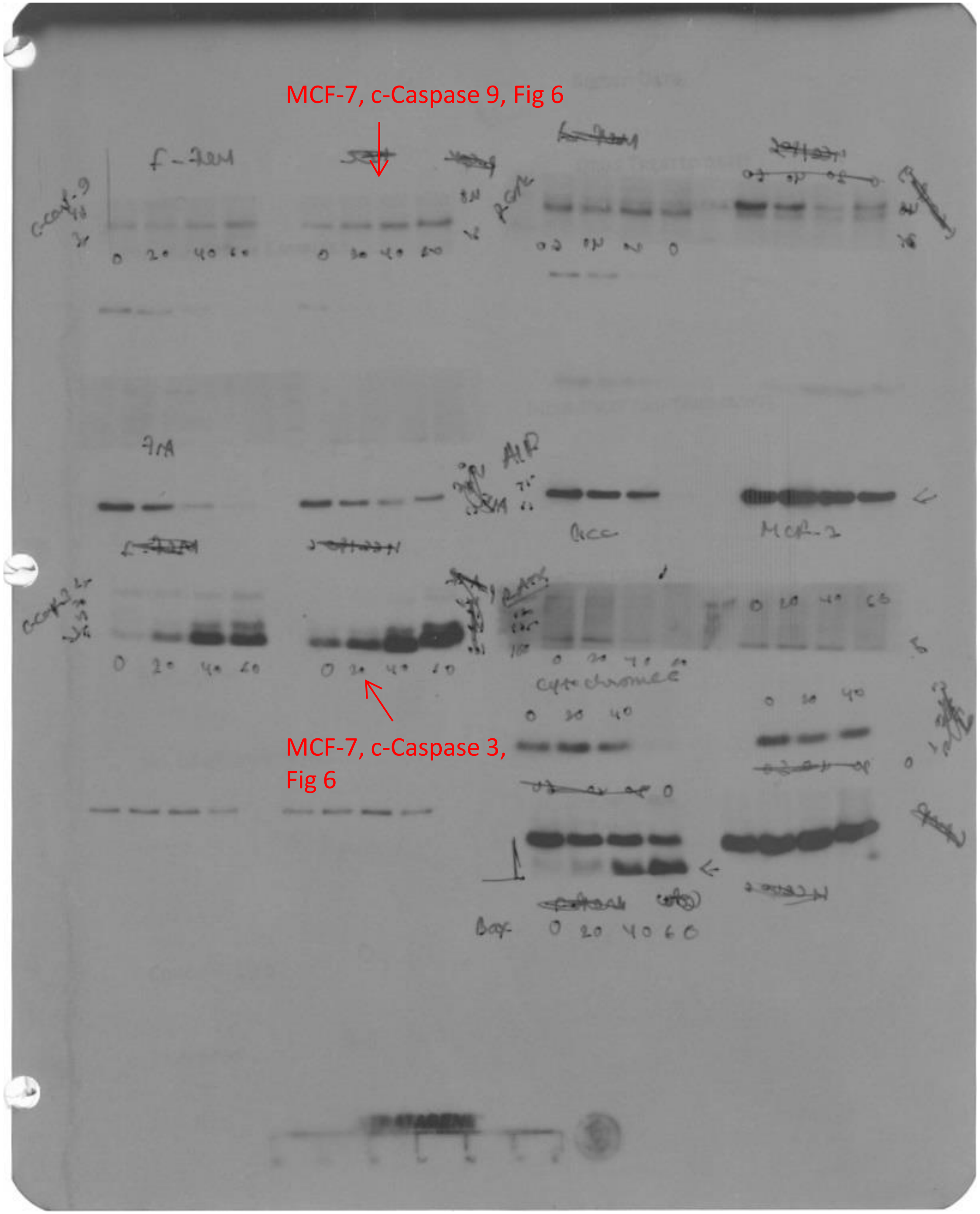
MCF-7, c-Caspase 9, Fig 6
MCF-7, c-Caspase 3, Fig 6

MDA-MB-231,
p-H2AX,Fig6

MDA-MB-231,
Bax,Fig6

MDA-MB-231,
c-Caspase 3,
Fig 6

MDA-MB-231,
c-Caspase 9,
Fig 6

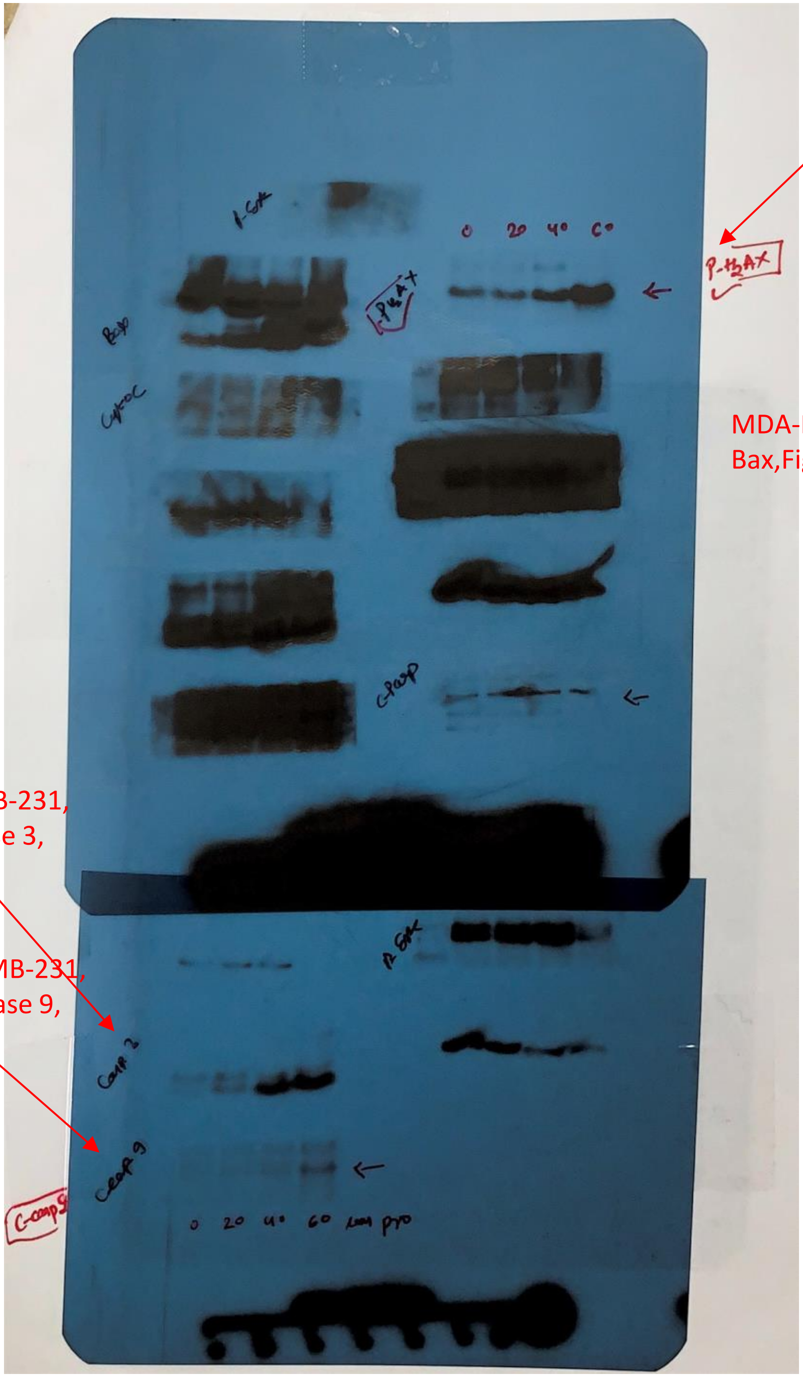

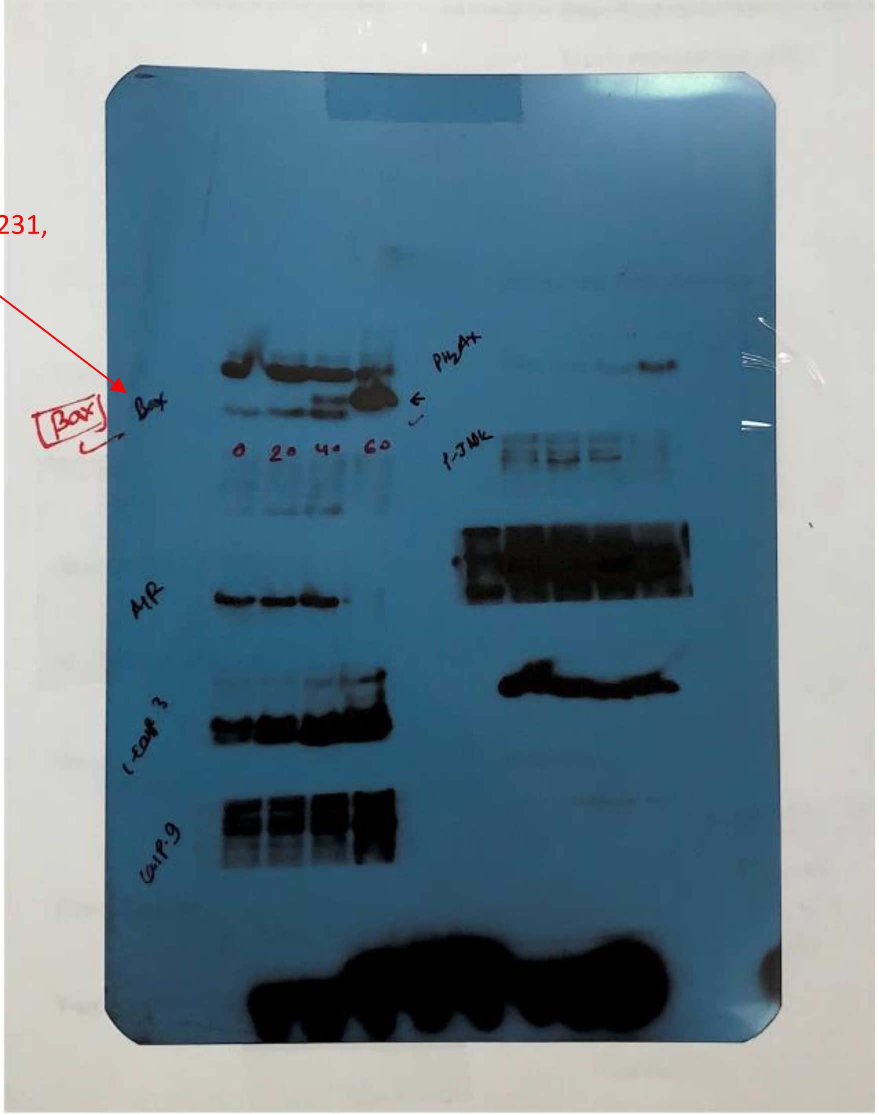

HCC1806, survivin ,Fig6

MDA-MB-231, survivin ,Fig6

MDA-MB-231, Bcl-2, Fig 6

HCC1806, Bcl-2, Fig 6

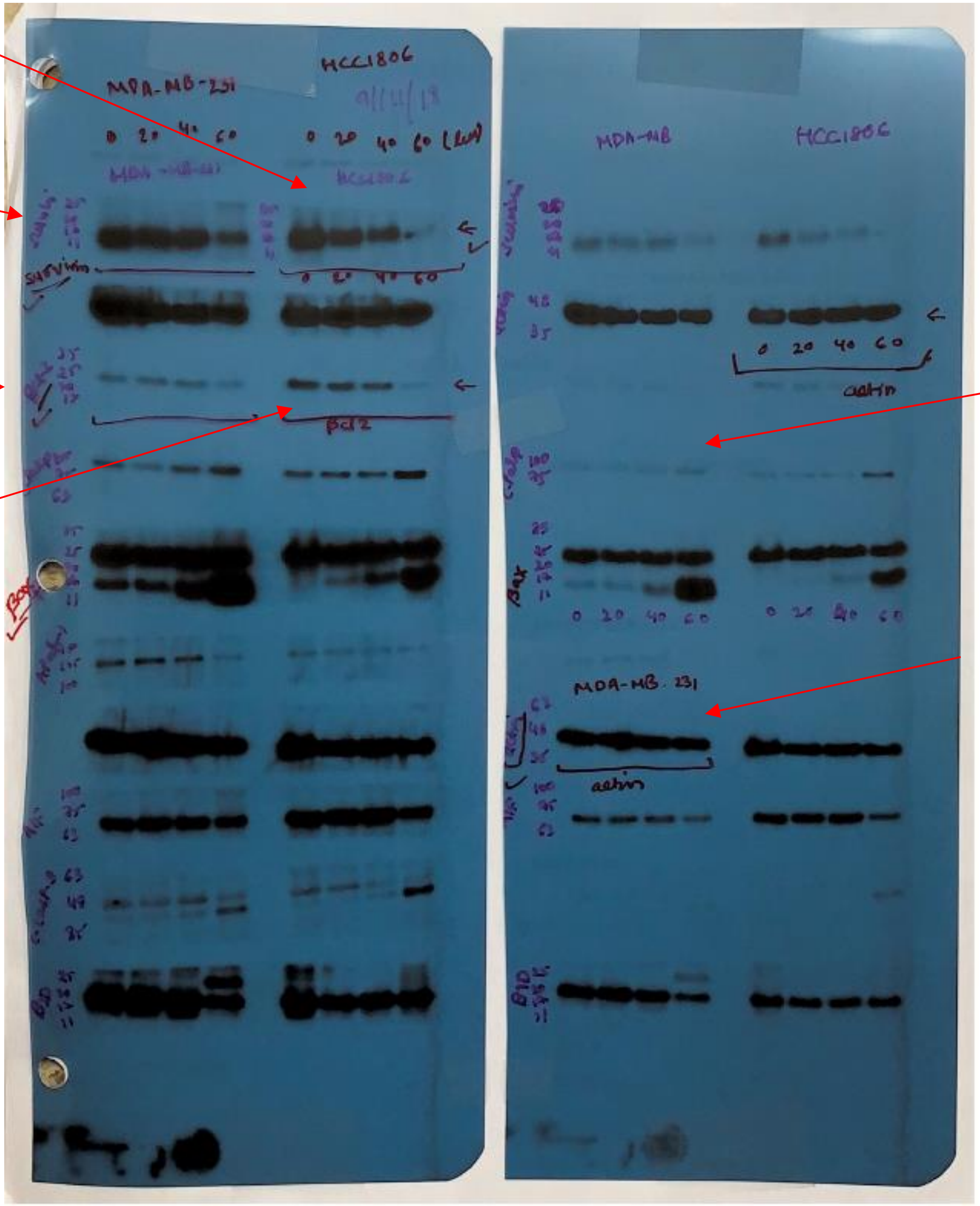

MDA-MB-231, c-PARP, Fig 6

MDA-MB-231, Actin, Fig 6

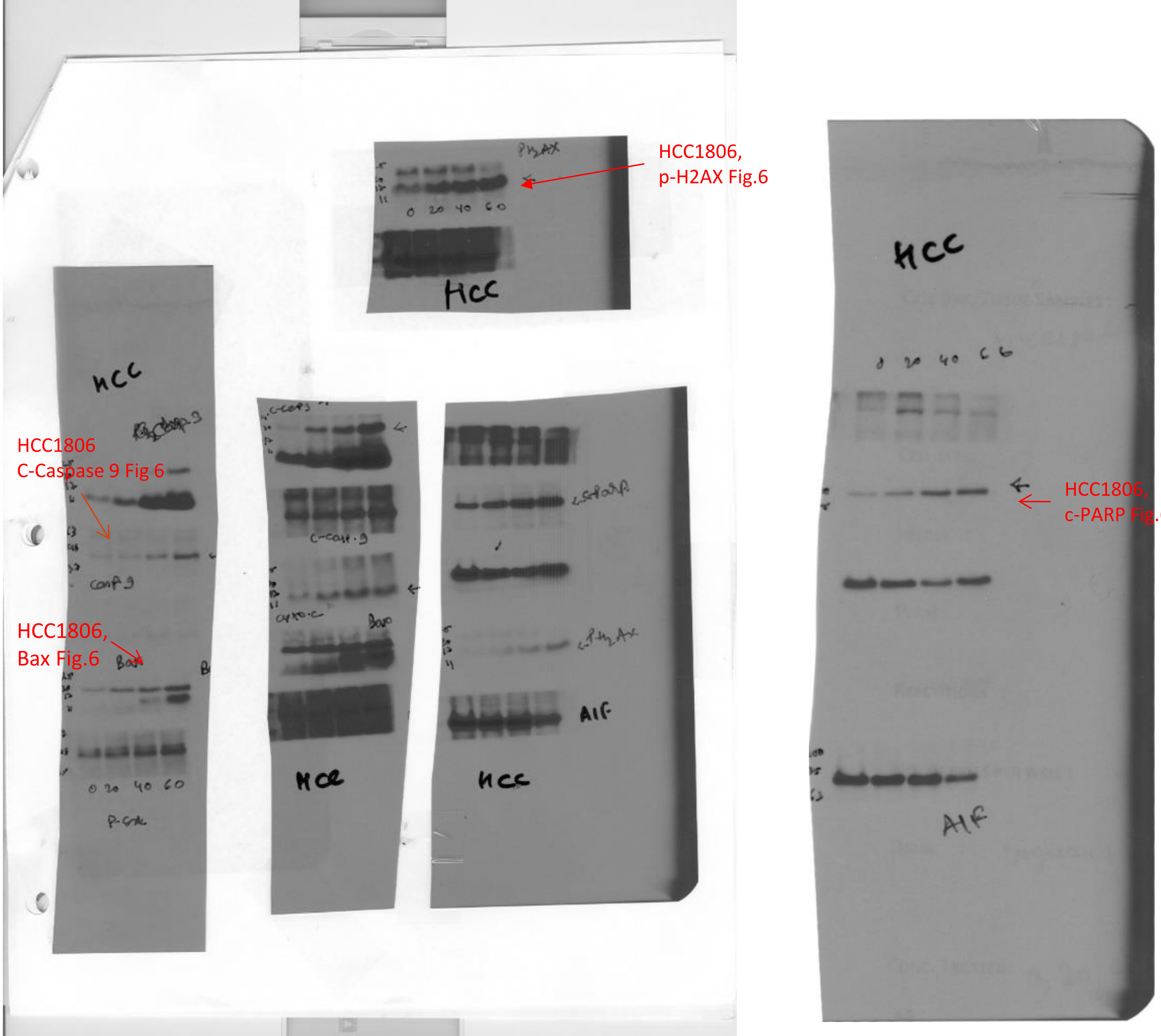
HCC1806, p-H2AX Fig.6
HCC1806 C-Caspase 9 Fig 6
HCC1806, Bax Fig.6
HCC1806, c-PARP Fig.6
HCC
0 20 40 60
AIF

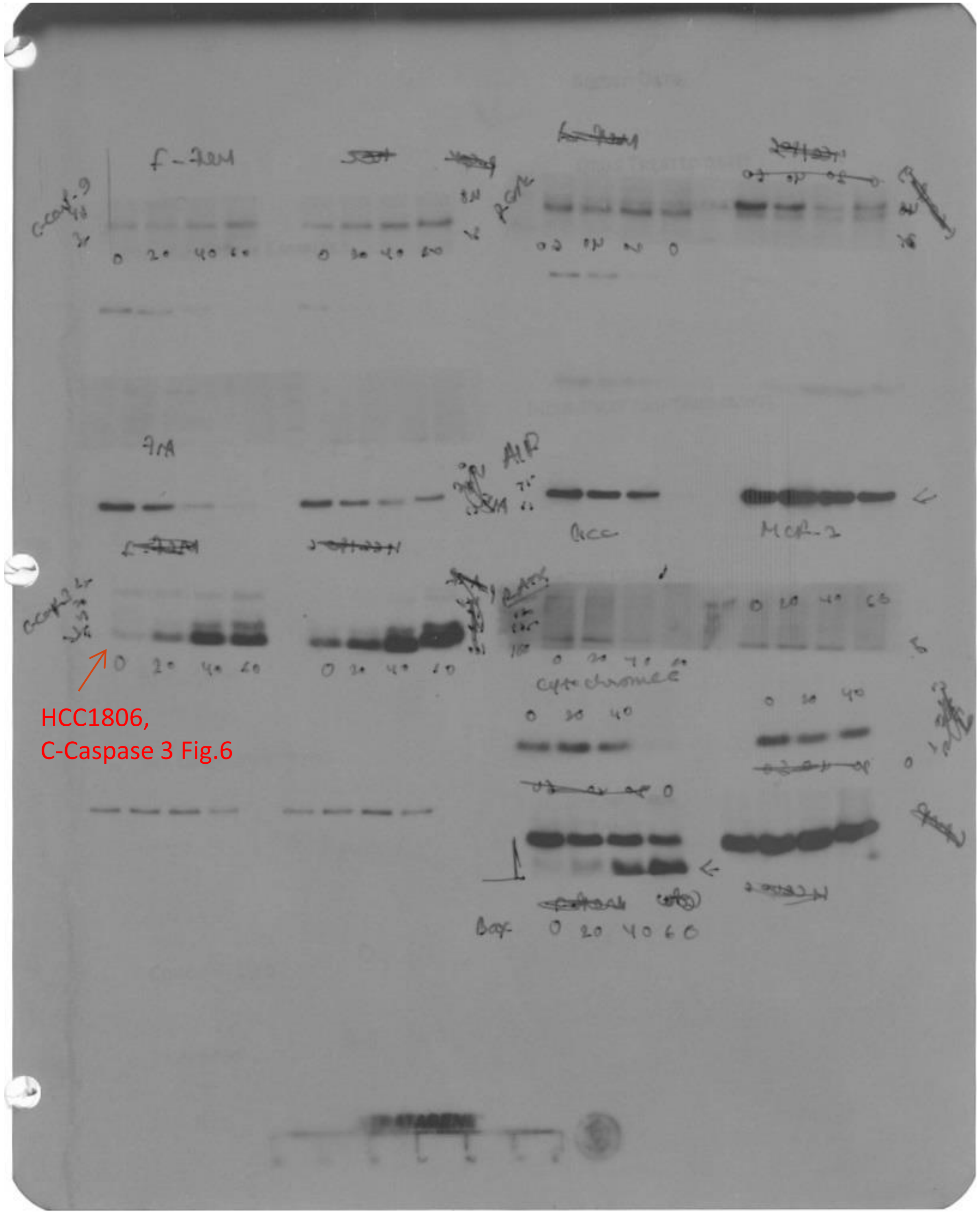
HCC1806,
C-Caspase 3 Fig.6

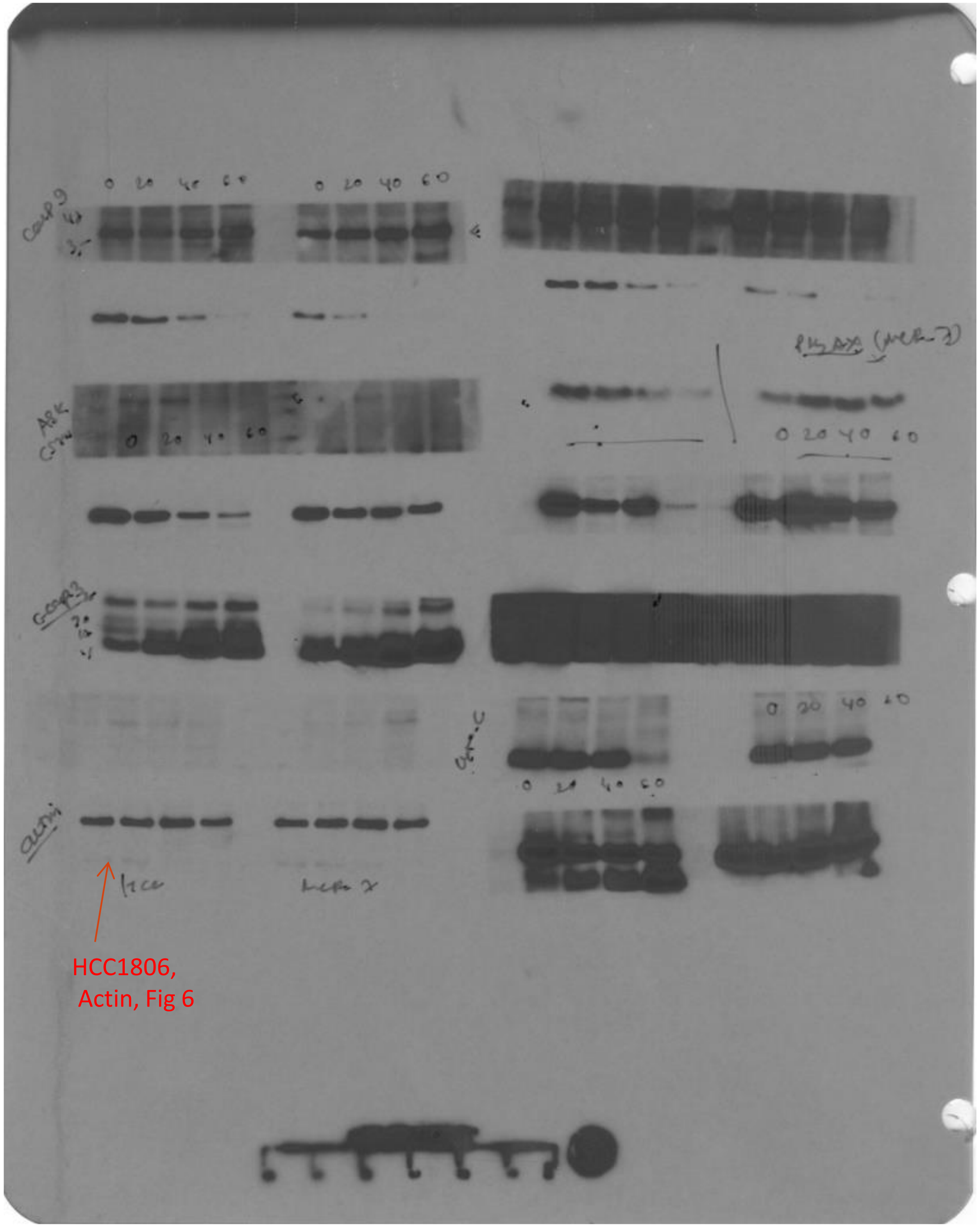
HCC1806,
Actin, Fig 6

Western blot raw data
Figure 9

4T1 Control, Cleaved Caspase-3, Fig 8

4T1 Treatment, Cleaved Caspase-3, Fig 8

4T1 Treatment, Actin, Fig 8

4T1 Control, Actin, Fig 8

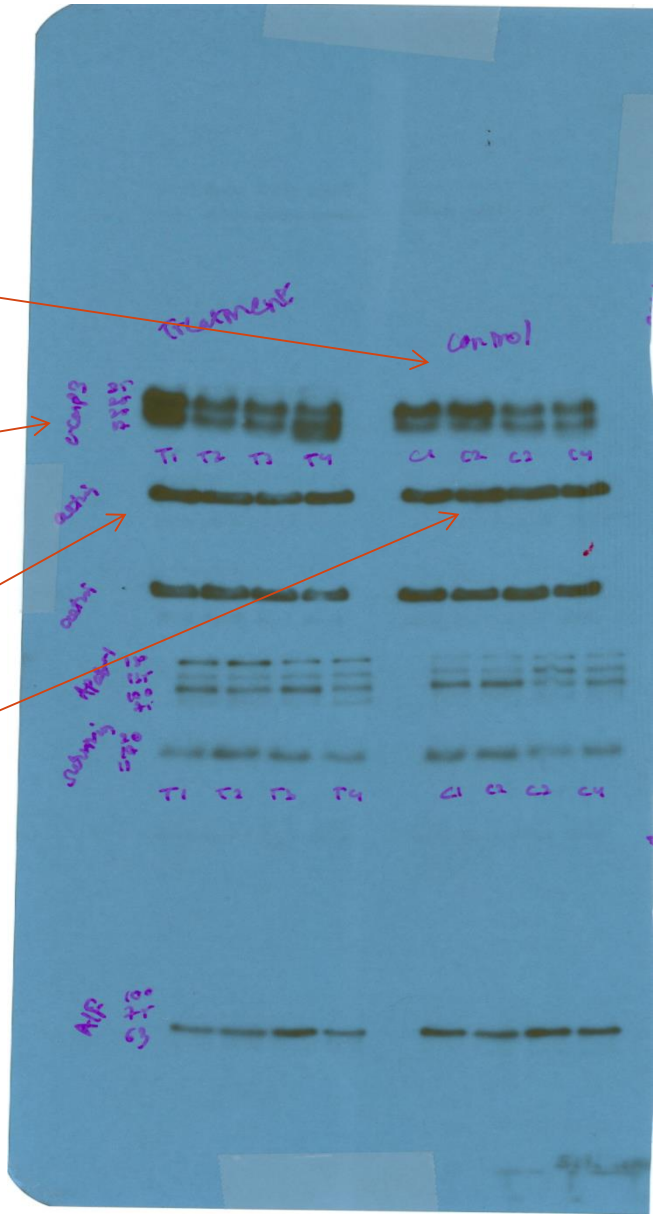

4T1 Control, C-PARP, Fig 8

4T1 Treatment, C-PARP, Fig 8

4T1 Treatment, BAX, Fig 8

4T1 Control, BAX, Fig 8

4T1 Treatment, P-H2AX, Fig 8

4T1 control, P-H2AX, Fig 8

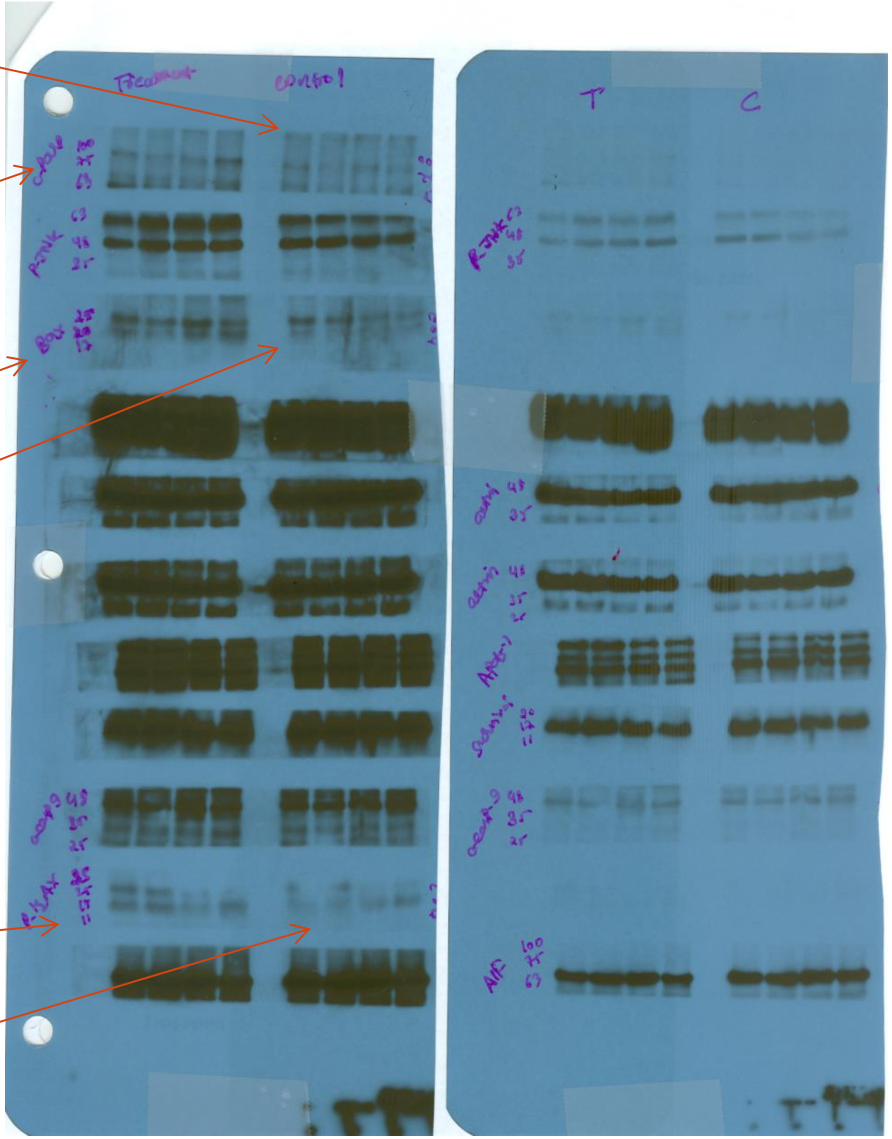

Supplement: Supplementary file 1 [file cancers-16-00872-s001.zip › cancers-2769389-supplementary.pdf]
